# The Feeding Rate of Predatory Mites on Life Stages of *Bemisia tabaci* Mediterranean Species

**DOI:** 10.3390/insects5030609

**Published:** 2014-07-22

**Authors:** Andrew G. S. Cuthbertson

**Affiliations:** The Food and Environment Research Agency, Sand Hutton, York YO41 1LZ, UK; E-Mail: andrew.cuthbertson@fera.gsi.gov.uk; Tel.: +44-0-1904-462201; Fax: +44-0-1904-462111

**Keywords:** *Bemisia tabaci*, predatory mite, Integrated Pest Management

## Abstract

The sweetpotato whitefly *Bemisia tabaci* (Gennadius) (Hemiptera: Aleyrodidae) continues to be a serious threat to crops worldwide. The UK holds Protected Zone status against this pest and, as a result, *B. tabaci* entering on plant material is subjected to a policy of eradication. There has recently been a shift from Middle East-Asia Minor 1 to the more chemical resistant Mediterranean species entering the UK. Predatory mites (*Amblyseius swirskii*, *Transeius montdorensis* and *Typhlodromalus limonicus*) were screened for their impact upon various lifestages of *B. tabaci* Mediterranean species. Approximately 30% of eggs were fed upon by *A. swirskii* following a 5 day period. Feeding rates slightly decreased for all mite species when feeding on first instar life-stages (27%, 24%, 16% respectively) and significantly decreased when feeding on second instars (8.5%, 8.5%, 8.7% respectively). Combining the two mite species (*A. swirskii* and *T. montdorensis*) increased mortality of *Bemisia* eggs to 36%. The potential of incorporating the mites into existing control strategies for *B. tabaci* is discussed.

## 1. Introduction

The whitefly *Bemisia tabaci* (Hemiptera: Aleyrodidae) is an obligate phloem-feeding pest, which is globally distributed, being found in all continents except Antarctica [1]. It affects more than 600 recognized plant hosts of both agricultural and horticultural crops and also ornamental plants [2,3,4]. *Bemisia tabaci* has been considered as one of the most destructive pests in tropical, subtropical and temperate zones [5,6]. Damage can be caused directly by feeding on phloem sap or indirectly by the large amounts of honeydew produced so lowering photosynthesis. *Bemisia tabaci* is also a vector of many plant viruses [7,8].

The pest status of *B. tabaci* insects is complicated by the recognition of 11 well-defined genetic biotypes and at least 24 morphocryptic species which are morphologically identical but distinguishable at the molecular level [6,9]. Through the comparison of mitochondrial cytochrome oxidase 1 (mtCO1) these 11 genetic biotypes can be mapped to Asia 1 (M, H); Australia/Indonesia, Australia (AN); China (Non B) Asia II (G, K, P); Asia II India, Italy (T); Sub-Saharan Africa non-silverleafing (E,S); New World (A, D); Africa/Middle East/Asia Minor (Q, J, L, Sub-Saharan Africa silverleafing, B, B2 and MS) and Uganda [6]. It is the Middle East-Asia Minor 1 and Mediterranean species that are the two most widely distributed, and as a result, best known species. These two species present the greatest threat to glasshouse crops worldwide [10]. The damaging Middle East-Asia Minor 1 (Biotype B) is described as an aggressive coloniser and is an effective vector of many viruses, whereas the Mediterranean species (Biotype Q) characteristically shows strong resistance to novel insecticides [11,12].

In relation to the UK, *B. tabaci* has been intercepted annually on imported plant material since 1987 [13]. The primary concern for the UK is that the whitefly imported on ornamental plants such as poinsettia (*Euphorbia pulcherrima*) can transfer and infect glasshouse salad crops such as tomato and cucumber with Tomato yellow leaf curl virus (TYLCV), Tomato yellow leaf curl Sardinia virus (TYLCSV) and Cucurbit yellow stunting disorder virus (CYSDV) all of which are not currently present in the UK. To-date, although there are many interceptions of *B. tabaci* in the UK, the species and its associated viruses have not become established; to this end the UK continues to hold “Protected Zone” status against this pest with statutory action aimed at eradication taken whenever it is found [13,14]. A recent study by Powell *et al*. [15] determined that there was a shift from Middle East-Asia Minor 1 to Mediterranean species entering the UK. Therefore, there is a need for various control options, including predatory mites, to be investigated against this *Bemisia* species. The aim of the current study was to determine the feeding rate of various commercially available predatory mites within the UK on *B. tabaci* Mediterranean in order to determine their potential to be incorporated into existing eradication strategies.

## 2. Experimental

### 2.1. Source of Insects

Specimens of *B. tabaci* Mediterranean were obtained from an outbreak situation in the UK during autumn 2012. They were then under quarantine conditions cultured in Perspex® cages (60 × 60 × 80 cm) on poinsettia plants following the method of Cuthbertson *et al*. [16]. Commercially available predatory mite species *Amblyseius swirskii*, *Transeius montdorensis* (still marketed in the UK as *Amblyseius montdorensis*) and *Typhlodromalus limonicus* were supplied by Syngenta Bioline, UK.

### 2.2. Feeding Trails

Following the method of Cuthbertson *et al.* [17], a single predatory mite was placed on poinsettia leaf discs, together with 10 *B. tabaci* eggs as prey. The eggs were transferred onto the leaf disc using a fine camel hairbrush. The leaf discs, eggs and mites were then contained in an experimental arena [18]. This briefly consisted of two acrylic plates sandwiching a filter paper wick that fed a square of blotting paper upon which was placed an excised cucumber leaf covered by a second (1 cm thick) acrylic sheet with a 5 cm diameter aperture drilled in it. The arenas were incubated in a controlled environment cabinet at 21 °C (average UK glasshouse temperature [19]), 65% relative humidity (r.h.), and 16:8 Light:Dark. The number of eggs that were attacked or had clear symptoms of feeding damage were recorded after five days to determine consumption rates. The experiment was replicated 20 times. The controls consisted of an identical experimental procedure to those used for each treatment, with the exception that a predatory mite was not placed in the experimental arenas.

The above procedure was repeated for first and second instar *Bemisia* life stages and for each mite species separately. A trial was also undertaken combining an individual *A. swirskii* and *T. montdorensis* together to investigate their combined predatory potential.

### 2.3. Data Analysis

Data was analysed where appropriate. Assuming normality and constant variance, analysis of variance (ANOVA) was used to test any significant difference between different treatments (mite species) and the control.

## 3. Results and Discussion

**Figure 1:**
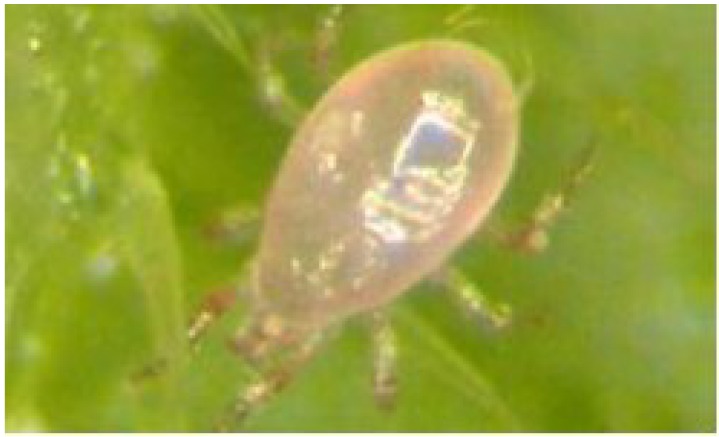
The predatory mite, *Amblyseius swirskii* (UK Crown Copyright^©^).

Phytoseiid mites are known to feed on phytophagous insects such as thrips [20] and whiteflies [21]. In particular, *A. swirskii* (Figure 1) has been shown to offer potential to be a major control agent against *B. tabaci* in several situations [22,23,24,25].

**Figure 2:**
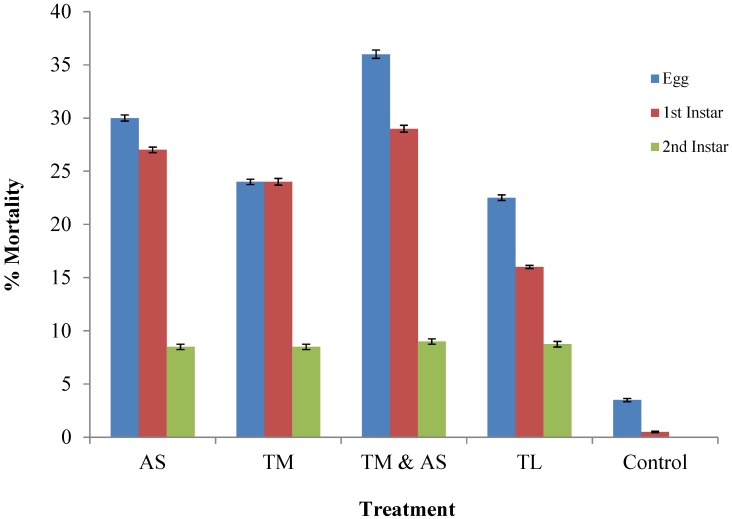
The impact of predatory mites against *Bemisia tabaci* Mediterranean lifestages. Mortality assessed following 5 days. AS—*Amblyseius swirskii*; TM—*Transeius montdorensis*; TL—*Typhlodromalus limonicus*.

Investigating the predation rates of the three predatory mite species (*A. swirskii*, *T. montdorensis*, *T. limonicus*) has proven all three readily feed upon the early life-stages of *Bemisia*. Approximately 30% of eggs were fed upon by *A. swirskii* following the 5 day period (Figure 2). There was no significant difference in egg consumption among the individual mites (*p* > 0.05). However, combining *T. montdorensis* and *A. swirskii* significantly increased egg consumption (*p* < 0.01). Predation percentages slightly decreased for all individual mite species when feeding on first instar life-stages (27%, 24%, 16% respectively) and significantly (*p* < 0.001) decreased when feeding on second instars (8.5%, 8.5%, 8.7% respectively).

In many cases, predatory mites have been successfully applied in the control of thrips but until relatively recent years no attempt has been made to use them in whitefly control [21]. Previous studies have shown the potential of predatory mites to be important components of integrated pest management strategies against the non-indigenous pest species *Thrips palmi* in the UK [17]. The predatory mites currently investigated show equal potential in regards to their efficacy against *B. tabaci* Mediterranean. Predatory mites are known to be most effective against egg and early instars of whiteflies [26], simply as adult whiteflies will fly away from an attacking predatory mite except during emergence from the last nymphal stage [27]. Combining the two species of mites gave some increase in mortality of all life-stages of *B. tabaci* tested against.

The predatory mites investigated are also known to show various levels of compatibility with various chemical insecticides [17]. They therefore offer potential to be incorporated into existing eradication strategies against *B. tabaci* in the UK.

## 4. Conclusions

*Bemisia tabaci* remains a severe threat to UK horticulture [13]. The continuing interception of insecticide resistance *B. tabaci* Mediterranean poses a real challenge in regards to maintaining the UK’s Protective Zone status. The mites investigated are known to have compatibility with several chemical insecticides [17] and so offer much potential to be incorporated into existing eradication strategies.
